# Optimized reagents for immunopotency assays on mesenchymal stromal cells for clinical use

**DOI:** 10.1007/s12026-023-09385-1

**Published:** 2023-04-29

**Authors:** Sílvia Torrents, Andrés Escudero del Moral, Margarita Codinach, Luciano Rodríguez, Sergi Querol, Joaquim Vives

**Affiliations:** 1 Banc de Sang i Teixits, Edifici Dr. Frederic Duran i Jordà, Passeig Taulat, 116, 08005 Barcelona, Spain; 2 Transfusion Medicine Group, Vall d’Hebron Research Institute, Universitat Autònoma de Barcelona, Passeig de La Vall d’Hebron 129-139, 08035 Barcelona, Spain; 3 Musculoskeletal Tissue Engineering Group, Vall d’Hebron Research Institute (VHIR), Universitat Autònoma de Barcelona, Passeig de La Vall d’Hebron 129-139, 08035 Barcelona, Spain; 4 Departament de Medicina, Universitat Autònoma de Barcelona, Passeig de La Vall d’Hebron 129-139, 08035 Barcelona, Spain

**Keywords:** Mesenchymal stromal cells, Immunomodulation, Quality & regulatory compliance, Batch release, Potency assay, Clinical use

## Abstract

**Abstract:**

Multipotent mesenchymal stromal cells (MSC) offer new therapeutic opportunities based on their ability to modulate an imbalanced immune system. Immunomodulatory potency is typically demonstrated in vitro by measuring the presence of surrogate markers (i.e., indoleamine-2,3-dioxygenase, IDO; tumor necrosis factor receptor type 1, TNFR1) and/or functional assays in co-cultures (i.e., inhibition of lymphoproliferation, polarization of macrophages). However, the biological variability of reagents used in the latter type of assays leads to unreliable and difficult to reproduce data therefore making cross-comparison between batches difficult, both at the intra- and inter-laboratory levels. Herein, we describe a set of experiments aiming at the definition and validation of reliable biological reagents as a first step towards standardization of a potency assay. This approach is based on the co-culture of Wharton’s jelly (WJ)-derived MSC and cryopreserved pooled peripheral blood mononuclear cells. Altogether, we successfully defined a robust and reproducible immunopotency assay based on previously described methods incorporating substantial improvements such as cryopreservation of multiple vials of pooled peripheral blood mononuclear cells (PBMC) from 5 individual donors that enable a number of tests with same reagents, also reducing waste of PBMC from individual donors and therefore contributing to a more efficient and ethical method to use substances of human origin (SoHO). The new methodology was successfully validated using 11 batches of clinical grade MSC,WJ. Methods described here contribute to minimize PBMC donor variability while reducing costs, streamlining assay setup and convenience and laying the foundations for harmonization of biological reagents usage in standardized immunopotency assays for MSC.

**Highlights:**

• The use of pools of peripheral blood mononuclear cells (PBMCs) in potency assays contributes to robust and reproducible results, which is key in the assessment of mesenchymal stroma cells (MSC) potency for batch release.

• Cryopreservation of PBMCs does not impact negatively on their activation and proliferation abilities.

• Cryopreserved pools of PBMC constitutes convenient off-the-shelf reagents for potency assays.

• Cryopreservation of pooled PBMCs from multiple donors is a way to reduce waste of donated PBMC and its associated costs, as well as reducing the impact of individual donor variability of substances of human origin (SoHO).

## Background

Regulatory development of advanced therapy medicinal products (ATMP), a new category of living medicines of complex biological nature including cell- and gene-based therapies, is hindered by the lack of harmonization of analytical methods for determination of their critical quality attributes (CQA), such as identity and potency [1, 2]. In addition to the lack of standardization, most potency assays fail to reflect the mechanism of action (MoA) of the proposed treatment, as a result of poor understanding of actual biological processes involved in their therapeutic activity in patients. In the case of multipotent mesenchymal stromal cells (MSC), significant efforts have been made by scientific societies to propose criteria for their identification regardless of the tissue source [3–5]. However, developers tend to adapt such criteria to their particular tissue sources of MSC, manufacturing processes, cell characterization methods, and estimated mechanisms of action, altogether resulting in different panels of CQA and specifications for MSC used in clinical trials [6–9]. Consequently, this situation raises concerns on comparability of MSC from different laboratories and even from different batches in the same production facility, therefore jeopardizing the validity of systematic reviews and meta-analyses. In this context, efforts to promote standardization would benefit the field by allowing for more meaningful comparisons among studies, thus allowing for a smoother clinical translation. It is therefore a pressing need to define and standardise potency tests as a first step for harmonization in the field [10]. Indeed replication as a result of standardization of methods and harmonization across laboratories would streamline research, gather relevant safety and efficacy data as well as allowing for cost savings [11]. In the present study, we defined and qualified reagents and then optimized methods for robust and reproducible immunopotency testing of MSC based on a previously described protocol by Oliver-Vila and collaborators [12], by incorporating substantial improvements such as cryopreservation of peripheral blood mononuclear cells (PBMC) pooled from several donors to enable multiple tests with the same batch of cellular reagents and therefore reducing the impact of individual donor variability on assay results.

## Results

### The effect of cryopreservation on PBMC

**Fig. 1:**
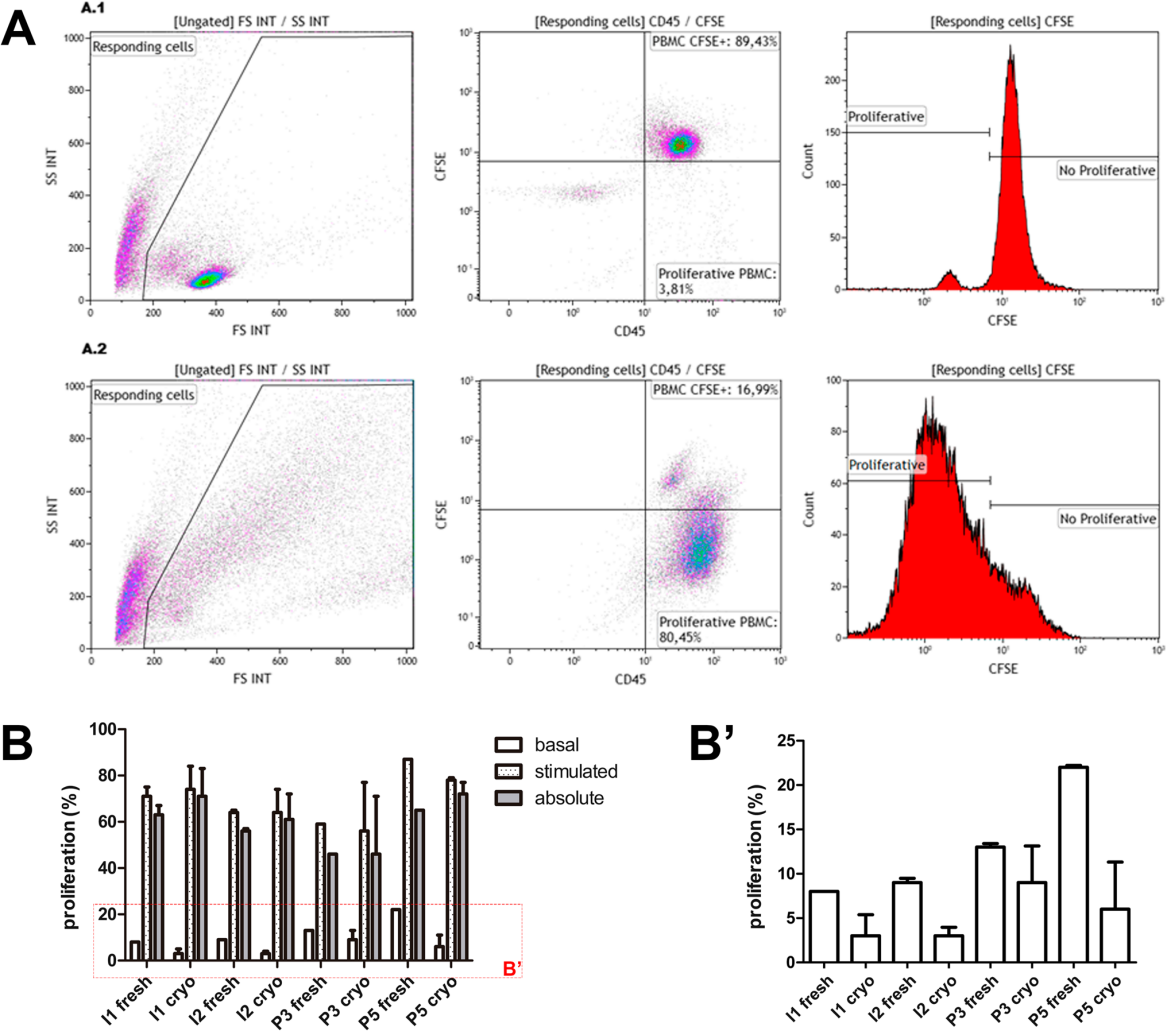
Proliferative capacity of cryopreserved individual and pooled PBMC preparations. Proliferative potential of PBMC (with or without stimuli) was determined by CFSE staining and flow cytometry analyses following the gating strategy shown in panel (**A**) Proliferative potential of two individual (I1 and I2) and two pooled PBMC preparations (pools of 3 and 5 donors, P3 and P5 respectively) were tested fresh and after thawing paired cryopreserved PBMC preparations (**B**) Basal proliferation of individual PBMC and pools is highlighted in B’ box, showing higher proliferation in pools both in fresh and after thawing cryopreserved PBMC. Absolute proliferation highlights the operating range for immunopotency assays

We first evaluated the impact of cryopreservation on cellular viability, cell yield and proliferation capacity by comparing individual and pool from 3 PBMC suspensions (9–13 mL per donor). To do this, the feasibility of using pools was tested on (i) fresh and cryopreserved buffy coats, (ii) investigating efficiency of carboxyfluorescein succinimidyl ester (CFSE) labelling in fresh and cryopreserved preparations (Fig. 1A), and (iii) evaluation of cell subpopulation composition. Although the presence of lymphocytes was relatively similar in fresh and thawed cryopreserved paired samples, some differences were observed regarding monocytes and granulocytes composition (Table 1). Despite using cryopreservation medium containing dimethyl sulfoxide, a cryoprotective agent that preserves most attributes of fresh samples, granulocytes were substantially reduced most probably due to granule release and/or granulocyte death (10.6% ± 6.0% vs. 2.4% ± 0.5% in fresh and cryopreserved PBMC from three individual donors; and 20.8% ± 23.2% vs. 8.0% ± 10.4% in fresh and cryopreserved PBMC from three pools). In all cases, CFSE labelling was ≥ 85% efficient.

**Table 1:** Comparison of composition and labelling efficiency in fresh and cryopreserved PBMC

%	CFSE labelling		Lymphocytes		Monocytes		Granulocytes	
	Fresh	Cryo	Fresh	Cryo	Fresh	Cryo	Fresh	Cryo
Individual PBMC	99.4 ± 0.4	99.9 ± 0.0	54.3 ± 3.1	57.4 ± 8.6	4.7 ± 2.2	6.3 ± 2.9	10.6 ± 6.0	2.4 ± 0.5
Pooled PBMC	99.5 ± 0.2	88.5 ± 0.5	57.4 ± 16.8	70.5 ± 7.9	10.6 ± 7.9	5.6 ± 5.3	20.8 ± 23.2	8.0 ± 10.4

Individual and pools of 3 PBMC preparations were analysed for composition (%) of main cell subpopulations as well as efficiency for carboxyfluorescein succinimidyl ester (CFSE) labelling (%), expressed as mean ± standard deviation (*n* = 3). Please note that data presented is not from a donor-matched study

*Cryo* cryopreserved

Considering that pooling 3 PBMC units was feasible, we also tested the possibility of using pools from 5 individual PBMC to generate more convenient volumes of reagents suitable for use in a test laboratory. Therefore, the first series of pools tested were composed of 3 and 5 buffy coats from different donors (Fig. 1). Regardless of using individual or pooled PBMC preparations, proliferation was clearly stimulated giving values of proliferating cells above 40% in all cases. Moreover, response of PBMC to polyclonal stimuli remained also above 40%, in all cryopreserved preparations (individual and pools) after thawing (Fig. 1).

The mixed lymphocyte reaction (MLR) observed in PBMC pools is the result of an allogeneic immune reaction activating T cells that occurs when lymphocytes of different donors are co-cultured without the need of external stimuli. In this sense, basal proliferation was lower in individual PBMC preparations compared to pooled PBMNC, as expected (Fig. 1B’). Despite of MLR, basal proliferation levels were significantly low (> 40% absolute proliferation in all cases) compared to the values achieved upon stimulation using strong activation stimuli (phorbol myristate acetate, PMA; and ionomycin) in all cases, therefore providing an ample range for the measurement of the inhibition of proliferation exerted by co-cultured MSC over background MLR (ranging from 46 to 65% and 46 to 72% absolute proliferation, in fresh and cryopreserved PBMC, respectively).

### Suitability of cryopreserved pools of PBMC in immunopotency testing of MSC

**Fig. 2:**
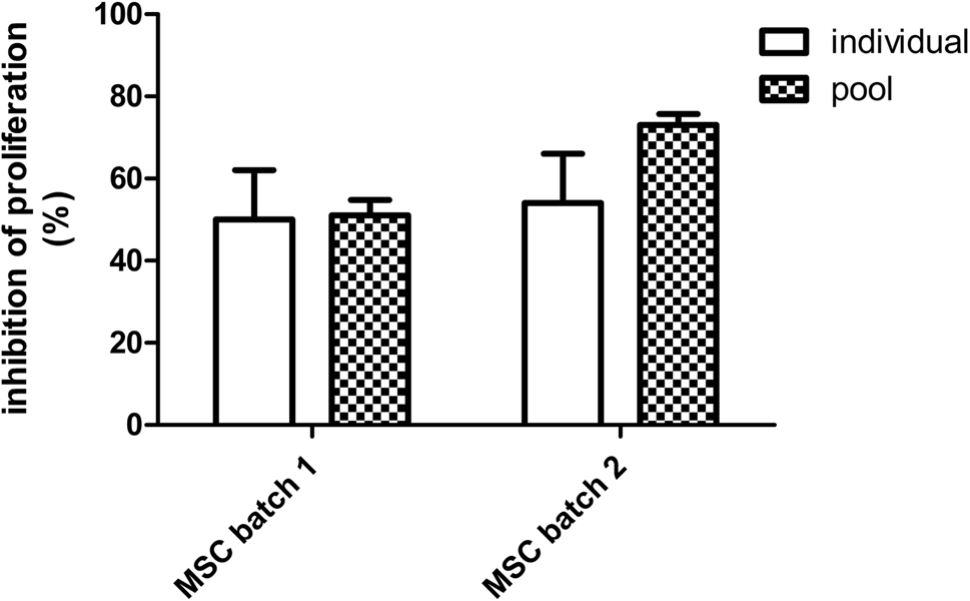
Inhibition of proliferation of two control MSC batches using individual and pooled PBMC preparations. Two independent lines of multipotent Mesenchymal Stromal Cells (MSC) were used for the assessment of immunopotency (expressed as percentage of inhibition of the proliferation of labelled PBMC) using individual PBMC preparations vs. a pool from three individual donors of PBMC (*n* = 3)

As presented previously, MLR is responsible of increased basal stimulation observed in pools compared to the use of individual donors (either fresh or cryopreserved). In order to determine whether MLR could interfere on the results of the potency assays, we tested two independent MSC cell lines using PBMC from individual donors and pooled PBMC (Fig. 2).

**Fig. 3:**
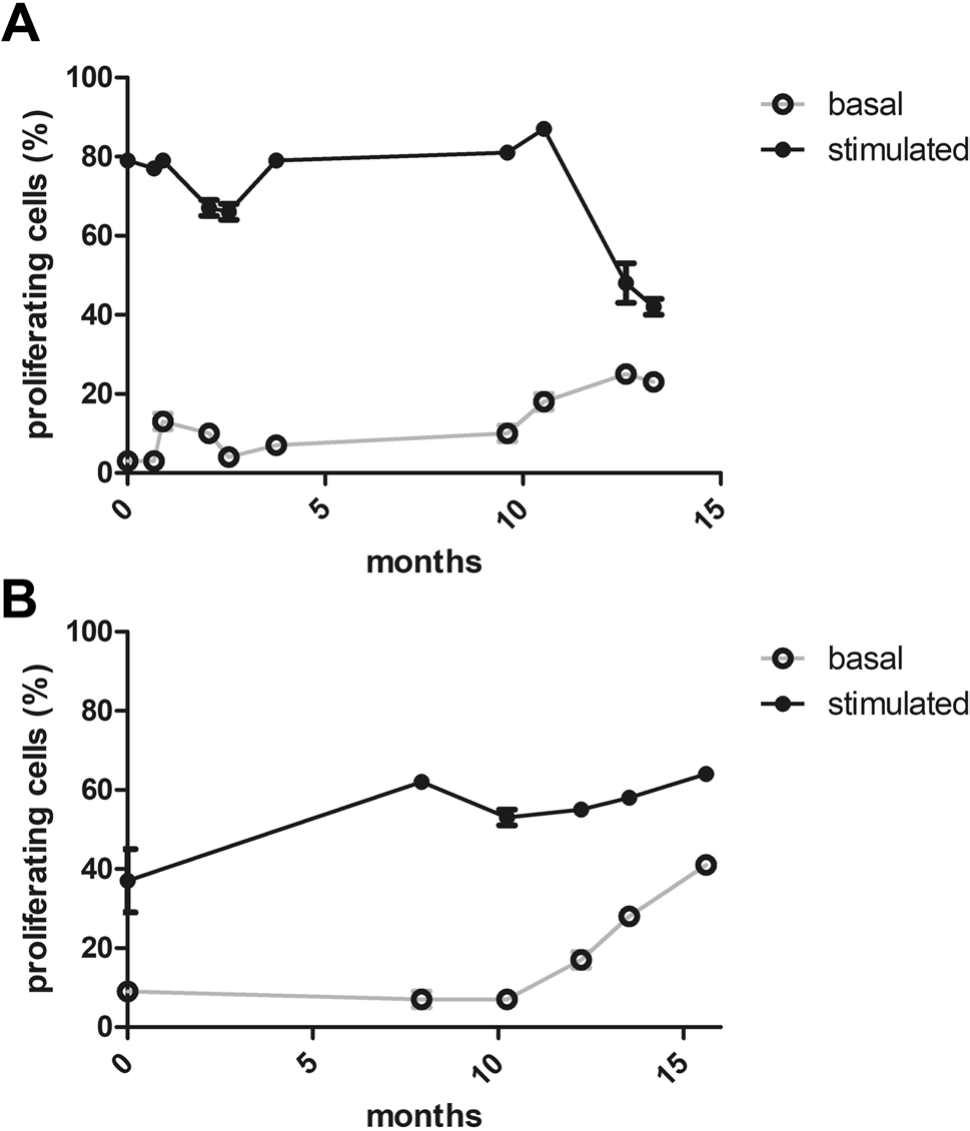
Stability of proliferation capacity of cryopreserved pools of PBMC. Cryovials from two pools of PBMC made from 5 donors each (in **A**, a pool composed of donor blood groups O^+^, A^−^, B^+^, A^+^, AB^+^; and in **B**, a pool composed of donor blood groups A^+^, O^+^, O^+^, O^+^, A.^+^) were thawed at different times (up to 13 and 15 months, respectively) and evaluated their capacity to proliferate in culture with and without polyclonal stimuli. Pool in **A** showed high capacity to respond to proliferation stimuli, whereas pool in B showed lower capacity to respond to proliferation stimuli. All measurements were performed on PBMC from cryovials thawed from either one of the two pools (**A** and **B**, respectively)

Remarkably, the use of pooled PBMC was found to be suitable for use in this type of assays. The resulting values of potency showed an acceptable range of variability, comprised either in the same range of those from individual donor PBMC for MSC line 1 (51% ± 3.8% vs. 50% ± 12%) or slightly higher when using pooled PBMC for MSC line 2 (73% ± 2.7% vs. 54% ± 12%) (Fig. 3). Importantly, in all cases, cells were found in conformity with the release criteria (> 30% inhibition of proliferation) established in the corresponding Investigational Medicinal Product Dossier (IMPD) of the approved MSC,WJ-based ATMP. Moreover, improved comparable inter-assay results are offered, which is key for the validation of MSC batches, provided that sufficiently large volumes of cryopreserved PBMC may be used for the assessment of immunopotency of different batches of MSC.

In order to determine whether MLR remained stable with time, we studied proliferation of PBMC in basal and stimulation conditions of cells previously cryopreserved in aliquots from two independent pools of 5 donors, which were thawed and used in immunopotency assays at different time points spanning more than 15 months (up to 399 days for pool A, and 468 days for pool B) (Fig. 3). For the two pools studied, the difference in the percentage of proliferating cells in the basal and stimulated conditions was very high for the first 12 months (average of 68%, ranging from 57 to 75%) and then reduced gradually at the two later time points (23% at 378 days and 19% at 399 days, respectively). Interestingly, despite the small differences (approximately 20%) of proliferating cells in the basal and stimulated conditions at later time points, we found that this range was sufficient to determine inhibitory properties of MSC,WJ on proliferation of PBMC in co-culture.

Taken together the data presented previously, we proposed pools from 5 individual donors that, in our hands, resulted in a volume > 10 mL at 6–8 × 10^7^ cells/mL, resulting in > 20 cryovials (composed of 0.5 mL of PBMC and 0.5 mL of cryopreservation solution). Although the inclusion of more donors would allow for a broader representation of population variability and provide standardised reagents for conducting a larger number of assays, it is also important to consider the burden of blood manipulation and the need to adjust to the anticipated number of assays in a given period of time (one year in total at 1–2 assays monthly, in our case) and stability (as shown in Fig. 3).

**Fig. 4:**
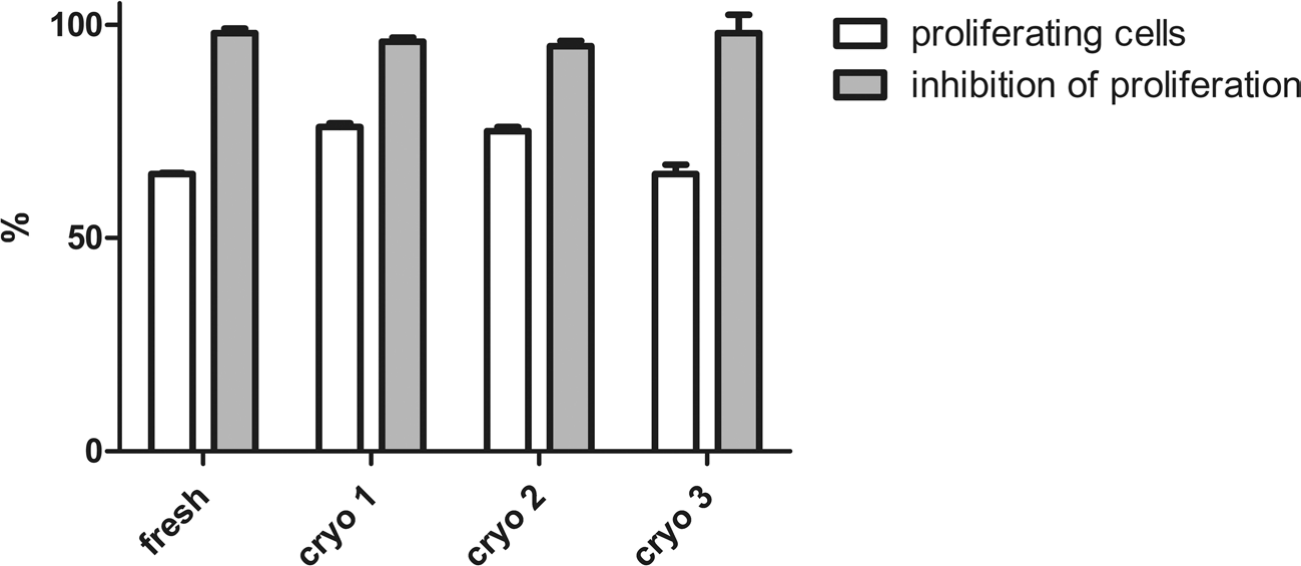
Proliferation of stimulated PBMC and inhibitory effect of MSC in co-culture. Although proliferation of stimulated PBMC ranged from 65 to 75%, inhibition of their proliferation by co-culturing with MSC,WJ was strong and showed a tight range between 95 and 98% in the three replicates performed (cryo 1, 2, and 3) and were comparable to the values resulting from the fresh control (98%)

Next, a control MSC line was used in four independent assays to validate repeatability and robustness, by conducting the assay at different time points (elapsed by 1–2 week each) in which PBMC stimulation and the inhibition of proliferation by MSC,WJ were assessed (Fig. 4). Reproducibility of our approach was assessed by thawing individual cryovials from the same PBMC pool and co-cultured with the same MSC line to evaluate the closeness of agreement between a series of measurements supporting inter-assay precision. Moreover, robustness was assessed by reproducing the method by different analysts (ST and AEdM) without the occurrence of unexpected differences in the obtained results.

The inhibition of proliferation by MSC,WJ remained constant in tests performed at different times with cryopreserved pools, also when compared to fresh PBMCs. Although variability was observed in terms of absolute proliferation (ranging from 65 to 75%), this had no impact on normalised values of inhibition of proliferation (Fig. 4). Indeed, variability in the results of the assay was kept in a tight range comprised between 95 and 98% when using pools.

### Implementation of cryopreserved pools of PBMC in routine release of MSC.WJ

**Fig. 5:**
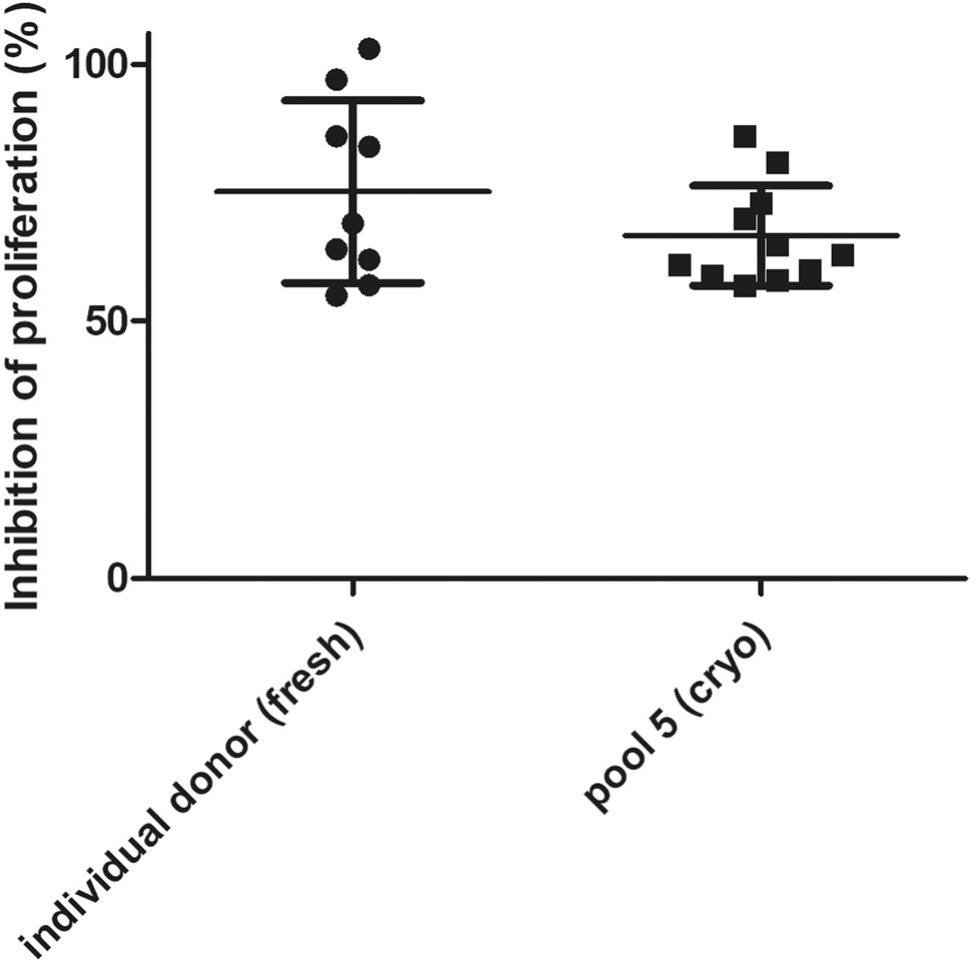
Comparability of pooled vs. individual PBMC in the assessment of immunopotency of clinical batches of MSC,WJ drug products derived from the same Master Cell Bank. Comparability study conducted on immunoptency assays of clinical-grade batches MSC,WJ used in the context of ongoing Phase I/IIa trials (EudraCT No.2021–000,346-18, Clinicaltrials.gov Id. NCT05054803; EudraCT No. 2020–001,505-22, ClinicalTrials.gov Id. NCT04390139; and EudraCT No. 2018–001,964-49, ClinicalTrials.gov Id. NCT03798353) and compassionate uses (in the management of graft versus host disease, GvHD). No statistically significant differences were observed between the potency assays performed on the group using individual donor of fresh PBMC (*n* = 9) and the group using cryopreserved pooled PBMC from 5 different donors (*n* = 11) (*P* = 0.1858; unpaired t test). Interestingly the range of inhibition values was smaller showing lower variability in the group using pooled PBMC

Finally, the success of the implementation of the improved methods for immunopotency assessment of MSC,WJ expanded from the same Master Cell Bank (same donor of umbilical cord) was evaluated using historical data of batches released from tests conducted using fresh preparations of PBMC from individual donors. Attributes of MSC,WJ are described in Table 2. These data were compared to the results of tests performed using cryopreserved pooled PBMC from 5 different donors following the methodology described previously. Interestingly we found no statistical differences (*P* = 0.1858) between the two groups, showing similar average values of inhibition of proliferation, being 75% ± 18% (*n* = 9) vs. 69% ± 13% (n = 11), respectively, while reducing variability of results (Fig. 5).

**Table 2:** Identity and immunopotency of clinical bacthes of MSC,WJ

*PBMC*	*MSC batch*	*CD45*^−^*/CD105*^+^ *(%)*	*CD31*^−^*CD73*^+^ *(%)*	*HLADR*^−^*/CD90*^+^ *(%)*	*CD90*^+^ *(%)*	*Inhibition of proliferation*
*Individual donors (fresh)*	1	99.9	99.8	99.4	99.9	62%
	2	99.9	99.9	99.3	99.7	97%
	3	99.9	99.9	99.5	99.8	84%
	4	100	99.9	99.5	99.7	103%
	5	99.7	99.4	99.2	99.7	64%
	6	99.9	99.7	99.5	99.9	69%
	7	100	99.9	998	99.9	86%
	8	99.7	99.8	99.8	99.9	57%
	9	99.7	99.9	99.8	99.9	55%
*Pooled from 5 donors (cryopreserved)*	10	99.8	99.9	99.9	100	86%
	11	99.5	99.8	99.6	99.8	81%
	12	99.1	99.8	99.9	100	70%
	13	100	99.8	99.8	100	59%
	14	100	99.5	99.6	100	61%
	15	99.8	99.6	99.9	100	58%
	16	99.4	99.7	99.6	100	73%
	17	98.7	99.8	99.6	99.8	57%
	18	98.7	99.6	99.6	100	63%
	19	97.6	99.7	99.2	99.9	60%
	20	99.8	99.6	99.4	100	65%

## Discussion

From all CQA commonly defined for MSC bioprocessing, those related to potency are mandatory for the release of clinically relevant batches as indicator of the product’s capacity to exert the desired therapeutic activity in patients [13, 14]. According to the International Council for Harmonisation of Technical Requirements for Pharmaceuticals for Human Use (ICH), potency is defined as the quantitative measure of biological activity based on attribute(s) of the product linked to its relevant biological properties [15]. Adequate potency assays are needed to predict the therapeutic efficacy of ATMPs, and these must be optimised and implemented throughout the product development programme and not only after marketing approval [16]. However, this endeavour is challenging due to our limited understanding of actual MoA in most clinical applications of MSC. Thus, both developers and regulators must agree on the choice of appropriate potency assays prior to clinical testing, providing sound justification of the scientific rationale and documented evidence [4, 17–20]. The establishment of suitable and reliable potency assays should guarantee batch-to-batch consistency for safe cellular products with the capacity to exert the intended therapeutic effect [21]. Given both our poor understanding of MoA and the lack of relevant and validated animal models (which are also associated to high costs and ethical concerns on their use), in vitro assays are preferred. Indeed, MSC immunopotency potential is commonly assessed in cell-based assays using PBMC or CD3^+^ selected populations.

Previously, we have proposed the use of fresh PBMC consisting of diverse cellular populations, mostly lymphocytes but also monocytes and granulocytes, reproducing to some extent the complexity of cell types that are found in the patient [12]. Although our approach to cryopreserve PBMC has shown to alter the representation of the granulocytic population, this did not impact on the measurements of inhibition of overall cell proliferation, because proliferating cells are polyclonally stimulated lymphocytes. Given that continuous improvements of potency assays must be done consistently according to scientific and technological progress, we believe that improvements of the protocol presented herein will help to overcome existing limitations in the variability of biological reagents for Quality Control (QC).

Importantly, we validated the new method in a clinically relevant MSC-based ATMP. Particularly, we focused our efforts to standardize methods for scale up production of multipotent MSC,WJ, as well as defining suitable QC assays to determine identity, purity and potency consistently as a pre-requisite for further clinical use in the context of inflammatory conditions, including spinal cord injury (EudraCT No. 2015–005,786-23, ClinicalTrials.gov Id. NCT03003364; and EudraCT No.2021–000,346-18, Clinicaltrials.gov Id. NCT05054803) and severe respiratory distress due to SARS-CoV-2 infection (EudraCT No. 2020–001,505-22, ClinicalTrials.gov Id. NCT04390139) [22–24]. Methods for measuring potency of MSC,WJ relied initially on individual blood donors for PBMC preparations but a pressing need to reduce variability of biological reagents prompted us to optimise the selection and their preparation. In addition to their differentiation potential, MSC are of clinical interest due to their ability to regulate inflammatory responses in diverse pathologies involving the immune system of the patient, being Graft versus Host Disease (GvHD) one of the best studied conditions [25], although the mechanisms of action and the role that these cells play in immunomodulation are still an open question without conclusive answers.

Altogether, our data demonstrates that pooled PBMCs are suitable for use in potency assays and may also contribute to the standardization of reagents for use in multiple assays intra- and/or inter-laboratory, therefore reducing the impact of variability of single-use reagents derived from different individual donors and thus making immunopotency assays more robust and reproducible, while making the entire process more ethically conscious by reducing waste of the unused individual fresh PBMC. Moreover, our results provide evidence that cryopreserved MSC hold immunomodulation capacity after thawing contributing to an open discussion in the scientific literature in which some authors propose an additional passage to enhance MSC’s therapeutic potential [7, 26–28].

## Conclusions

Cryopreserved pooled PBMC offer a reliable source of biological reagents for consistent and reproducible assessment of immunopotency by using an improved protocol of lymphoproliferation assay that was successfully validated using GMP-grade MSC,WJ.

## Methods

### Overall procedure details

**Fig. 6:**
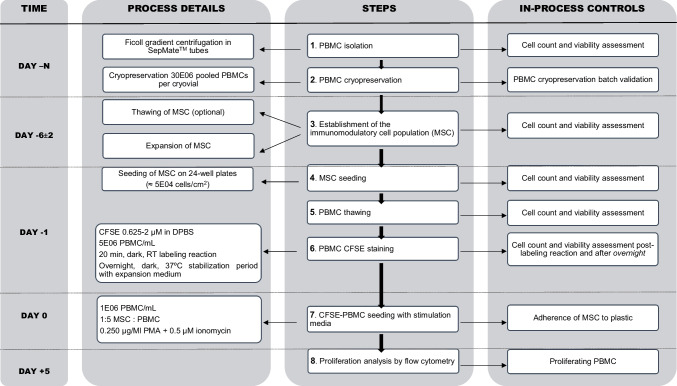
Flowchart. Sequential steps, time considerations, and in-process controls

The optimised protocol developed in this work is presented in a flowchart including sequential steps and in-process controls (Fig. 6). Specific details are describe in the next subsections.

### Cell sourcing, MSC derivation, and expansion

PBMC were obtained by a density gradient centrifugation (Histopaque-1077; Sigma-Aldrich, Saint Louis, MO, USA) from 24- to 48-h-old buffy-coats of healthy blood donors aged 18–70 years old, which were confirmed negative for serology markers: HBsAb, HIV I/II, Lues (TPHA), Chagas, HBcAb, HCV, anti-HTLV I/II, NAD (HCV-HIV, HBV). PBMCs were used individually or pooled, fresh or cryopreserved, according to the experimental design. MSC were derived from the Wharton’s Jelly (WJ) of donated umbilical cords following GMP-compliant procedures reported elsewhere [29, 30], with appropriate donor informed consent. After the isolation step, cells were replated by seeding T cell culture flasks at 1.5–3 × 10^3^ cell/cm^2^. When the total number of viable cells reached at least 5 × 10^6^, they were cryopreserved as master cell bank (MCB). Further expansion was performed after thawing vials of MCB using expansion medium composed of DMEM containing 2 mmol/L glutamine and supplemented with 10% pooled inactivated human serum B (hSerB; Banc de Sang i Teixits, Barcelona, Spain) [13, 31]. All cultures were maintained at 37 °C and 5% CO_2_ in humidified incubators. Media were replaced every 3–4 days, and trypsinization was performed at 70–90% confluence.

### Cryopreservation

PBMC were collected from Ficoll gradient centrifugation of approximately 15 mL of buffy coat from each donor in Sepmate tubes (StemCell Technologies, Vancouver, CA). Next, pools were prepared and cells were cryopreserved in a solution composed of Dulbecco’s Phosphate-Buffered Saline (DPBS; Gibco) supplemented with 10% (v/v) dimethyl sulfoxide (DMSO; OriGen Biomedical, Austin, TX, USA) and 2% (w/v) human serum albumin (HSA; Grifols, Barcelona, Spain), by applying a controlled freezing rate of 1 °C/min in a Mr. Frosty device (Nalgene, Rochester, NY, USA) kept in a − 80 °C freezer for 24 h before storage at − 196 °C in a liquid nitrogen tank until further use [32]. When needed, typically within 28 days at the times shown in Fig. 3) cells were rapidly thawed in a 37 °C water bath, then slowly diluted 1:10 using pre-cooled thawing solution consisting of 10% (w/v) albumin in Plasmalyte 148. DMSO was washout by centrifugation at 340* g* for 10 min and the cell pellet was resuspended in appropriate culture medium according to the experimental design.

### Flow cytometry

Cells were enumerated using Perfect-Count Microspheres™ (Cytognos, Salamanca, Spain) microbeads in a Navios EX flow cytometer (Beckman Coulter, Pasadena, CA, USA). Viability (%) was determined using the 7-Amino-Actinomycin D (7-AAD, Beckman Coulter, Pasadena, CA, USA) exclusion method. Data were analyzed with Navios EX Software v2.0 (Beckman Coulter) software. In accordance with de International Society on Cell and Gene Therapy (ISCT) criteria [3], identity of MSC was evaluated by the expression of surface markers CD31 PacificBlue-conjugated (clone 5.6E; Beckman Coulter), CD45 KromeOrange-conjugated (clone J33; Beckman Coulter), CD73 PE-conjugated (clone AD-2; Beckman Coulter), CD90 FITC-conjugated (clone F15-42–1-5, Beckman Coulter), CD105 PC7-conjugated (clone TEA3/17.1.1; Beckman Coulter), and HLA-DR APC-conjugated (clone Immu-357; Beckman Coulter) in a Navios EX Device. Cells were stained for 15 min at room temperature, washed and resuspendend with DPBS. Acquisition was done using Navios EX, and raw data were analyzed with Navios EX Software (version 2.0, Beckman Coulter).

### Lymphocyte proliferation assay

The immunomodulation potential of MSC,WJ was determined by their capacity to inhibit the proliferation of polyclonally stimulated lymphocytes in vitro, following an improved method based on that originally described by Oliver-Vila and collaborators [12], as described next. Briefly, 2.5 × 10^6^ PBMC/mL were labelled with 0.625 μM carboxy–fluorescein diacetate succinimidyl ester (CFSE) for 20 min at 37ºC using the CellTrace™ CFSE Cell Proliferation Kit (Molecular Probes, Eugene, OR, USA). After washing, (1, 2) × 10^7^ cells/mL were incubated for 12 min at 37 °C, washed again and seeded onto flat-bottomed 24-well plates (Corning, Corning, NY, USA) at 5:1 PBMC:MSC,WJ ratio. Typically, MSC,WJ cultures required 1–2 passages upon thawing GMP grade cryovials. Lymphocytes were activated with 25 ng/mL Phorbol 12-myristate 13-acetate (PMA, Sigma-Aldrich) and 0.5 μM Ionomycin (Sigma-Aldrich) in a final volume of 0.5 mL/well of DMEM containing 2 mmol/L glutamine and supplemented with 10% hSerB. Proliferation of PBMC was determined by measuring the reduction of fluorescence intensity at day 5 by flow cytometry using a Navios EX, and raw data were analyzed with Kaluza Analysis Software (version 2.1, Beckman Coulter). Inhibition of PBMC proliferation was calculated using Eqs. 1, 2 and 3, as described next:

1 $$absolute\;proliferation\;=\;\overline{stimulated\;}-\overline{nonstimulated\;}$$

2 $$normalized\;proliferation\;\left(\mathrm{\%}\right)\;=\;\frac{\mathrm{absolute\;proliferation\;w}/\mathrm{\;MSC}}{\mathrm{absolute\;proliferation\;w}/\mathrm{o\;MSC}}\;x\;100$$

3 $$Inhibitition\left(\mathrm{\%}\right)\;=\;100\;-\;normalized\;proliferation\;of\;co-culture$$

### Statistical analysis

Descriptive data were expressed as mean ± standard deviation (number) or range, as detailed in the text. Statistical significance was assessed by the *t*-test as described in the text and figure legends using GraphPad Prism (GraphPad software Inc., La Jolla, CA).

## Data Availability

All data generated or analyzed during this study are included in this published article and its additional file.
